# Crystal structure and Hirshfeld surface analysis of a new benzimidazole compound, 3-{1-[(2-hy­droxyphen­yl)meth­yl]-1*H*-1,3-benzo­diazol-2-yl}phenol

**DOI:** 10.1107/S2056989023010368

**Published:** 2024-01-01

**Authors:** Zakaria Bouhidel, Kaouther Sahli, Aouatef Cherouana

**Affiliations:** a Unité de Recherche de Chimie de l’Environnement et Moléculaire Structurale (URCHEMS), Département de Chimie, Université Mentouri de Constantine, 25000 Constantine, Algeria; b Pharmaceutical Sciences Research Center CRSP, Constantine 25000, Algeria; Venezuelan Institute of Scientific Research, Venezuela

**Keywords:** benzimidazole, single crystal, X-ray diffraction, hydrogen bonding, inter­mol­ecular inter­actions, Hirshfeld surface analysis

## Abstract

The title compound, C_20_H_16_N_2_O_2_, is composed of two monosubstituted benzene rings and one benzimidazole unit. The benzimidazole moiety subtends dihedral angles of 46.16 (7) and 77.45 (8)° with the benzene rings, which themselves form a dihedral angle of 54.34 (9)°. The crystal structure features O—H⋯N and O—H⋯O hydrogen-bonding inter­actions, which together lead to the formation of two-dimensional hydrogen-bonded layers parallel to the (101) plane. In addition, π–π inter­actions also contribute to the crystal cohesion. Hirshfeld surface analysis indicates that the most significant contacts in the crystal packing are: H⋯H (47.5%), O⋯H/H⋯O (12.4%), N⋯H/H⋯N (6.1%), C⋯H/H⋯C (27.6%) and C⋯C (4.6%).

## Abstract 2

In the title compound, the benzimidazole moiety subtends dihedral angles of 46.16 (7) and 77.45 (8)° with the benzene rings, which themselves form a dihedral angle of 54.34 (9)°. The crystal structure features O—H⋯N and O—H⋯O hydrogen-bonding inter­actions, which together lead to the formation of two-dimensional hydrogen-bonded layers parallel to the (101) plane, as well as π–π inter­actions

## 1. Chemical context

The benzimidazole unit comprises a phenyl ring fused to an imidazole ring. The first benzimidazole compound was prepared by Hoebrecker (1872 ▸). Benzimidazole is an important structural core in medicinal chemistry and this class of compounds displays a broad range of biological activities such as anti­microbial, anti­viral, anti­cancer, anti-inflammatory, gastroprotective and analgesic (Spasov *et al.*, 1999 ▸; Sevak *et al.*, 2002 ▸; Demirayak *et al.*, 2005 ▸). The use of benzimidazole derivatives with common drugs employed in the treatment of giardiasis has been reviewed (Harris *et al.*, 2001 ▸). The coord­ination behavior of benzimidazole derivatives towards trans­ition-metal ions was explored in order to increase their biological activity (Téllez *et al.*, 2007 ▸). The present work describes the synthesis, structural characterization and Hirshfeld analysis of a new benzimidazole compound, 3-{1-[(2-hy­droxy­phen­yl)meth­yl]-1*H*-1,3-benzo­diazol-2-yl}phenol. 
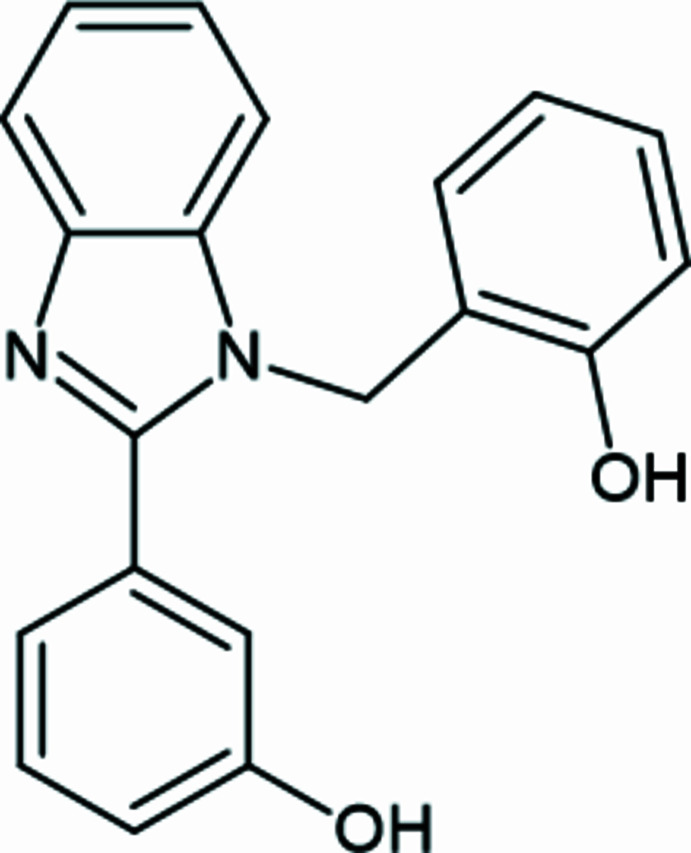


## 2. Structural commentary

**Figure 1:**
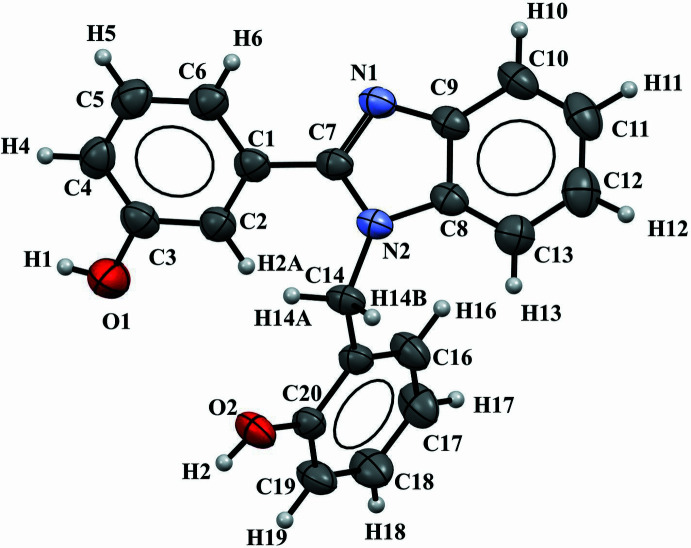
*ORTEP* view of the title compound with displacement ellipsoids drawn at the 50% probability level.

The title compound is composed of two monosubstituted benzene rings and one benzimidazole unit (Fig. 1 ▸). The benzimidazole moiety subtends dihedral angles of 46.16 (7) and 77.45 (8)° with the benzene rings, which themselves form a dihedral angle of 54.34 (9)°. These angles are in agreement with those observed in similar structures (Quezada-Miriel *et al.*, 2012 ▸; Shu-Ping Yang *et al.*, 2007 ▸).

## 3. Supra­molecular features

**Figure 2:**
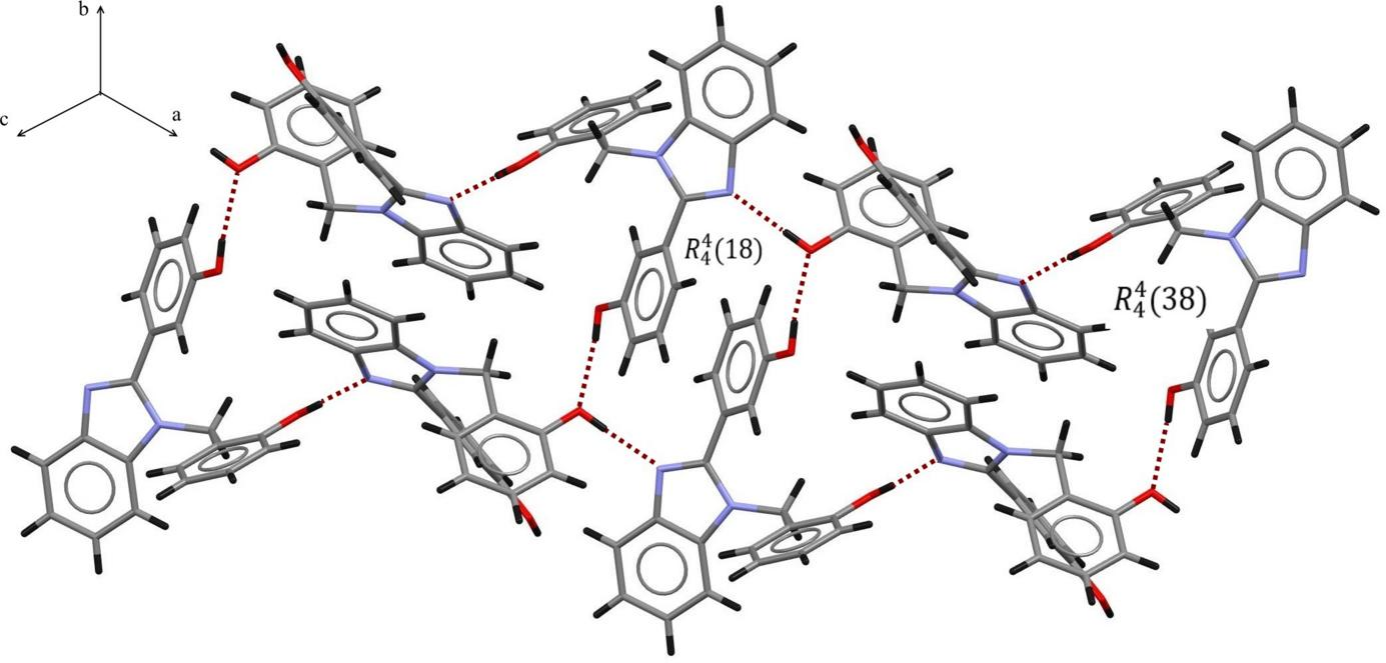
The 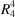 (18) and 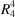 (38) graph-set motifs parallel to the *ab* plane generated by the combination of O—H⋯O and O—H⋯N hydrogen bonds.

The crystal packing of the title compound reveals inter­molecular hydrogen bonding, specifically O—H⋯O inter­actions involving benzene rings and O—H⋯N interaction between the benzimidazole moieties and benzene rings (Table 1 ▸). The mol­ecules are self-assembled by inter­molecular hydrogen bonds between the hydroxyl groups and the N1 atoms of the benzimid­azole moieties, forming hydrogen-bonded ribbons with a *C*(8) graph-set motif (Etter *et al.*, 1990 ▸; Bernstein *et al.*, 1995 ▸) parallel to the (111) plane. The combination of the O—H⋯O and O—H⋯N hydrogen bonds leads to rings with 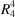 (18) and 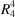 (38) graph-set motifs (Fig. 2 ▸). Further cohesion of the crystal packing is provided by π–π stacking inter­actions between C1–C6 benzene rings with centroid–centroid distances of 3.5957 (11) Å.

## 4. Hirshfeld surface analysis

**Figure 3:**
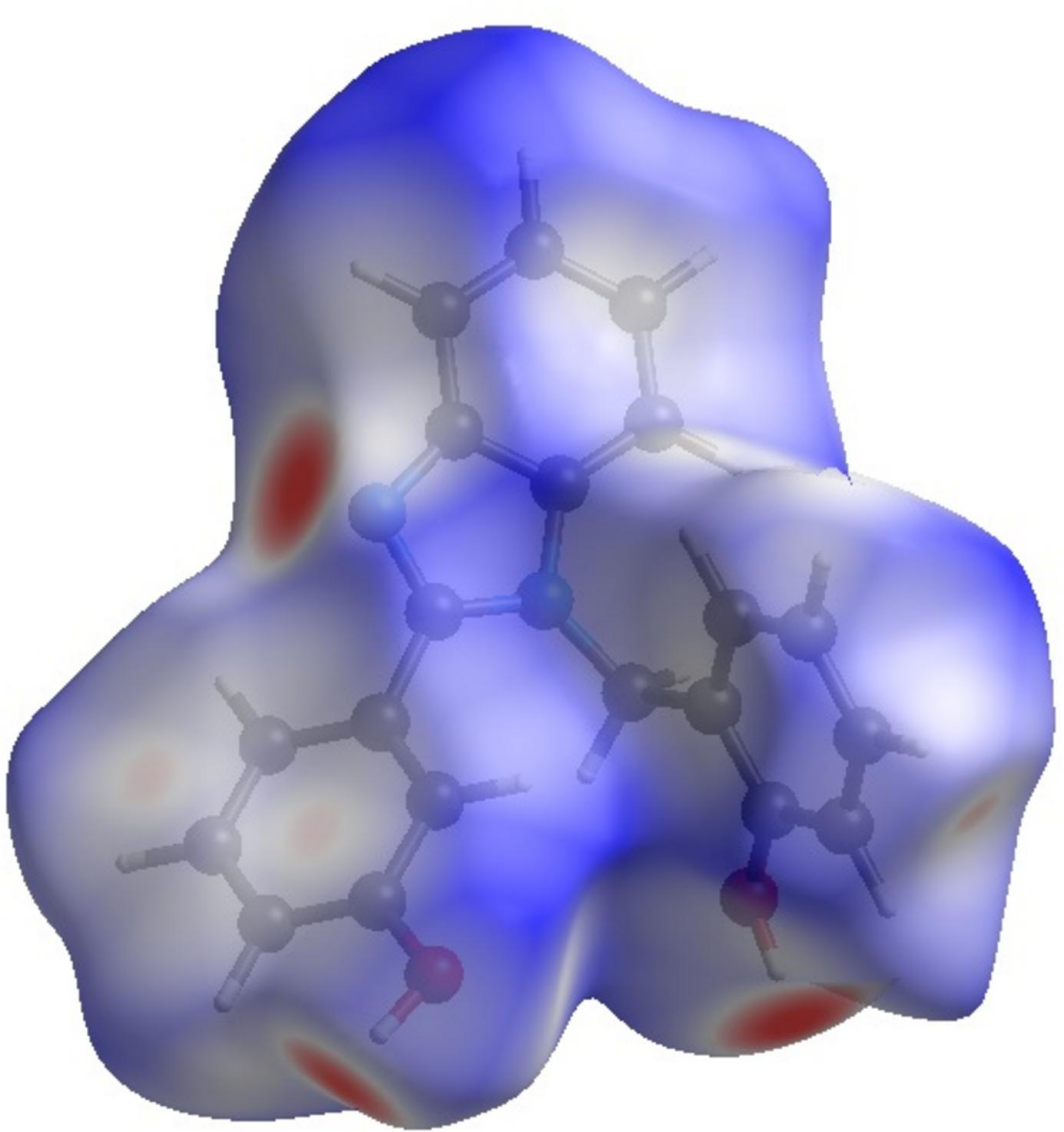
Hirshfeld surface of the title compound mapped with *d*_norm_.

**Figure 4:**
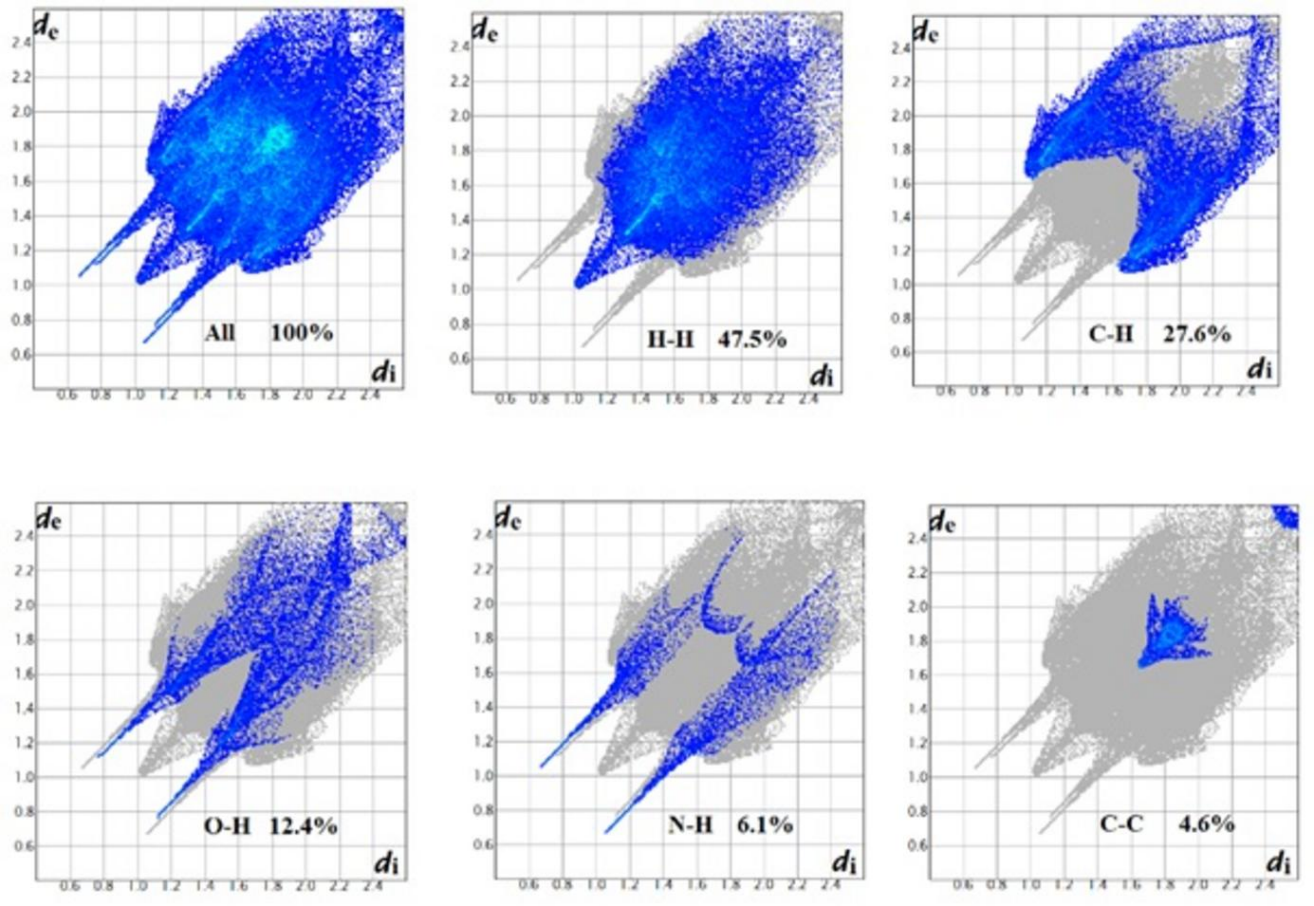
Two-dimensional fingerprints plots of the title compound, showing H⋯H, C⋯H/H⋯C, O⋯H/H⋯O, N⋯H/H⋯N and C⋯C contacts.

**Figure 5:**
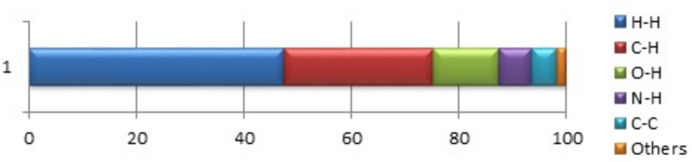
Relative contributions of various inter­actions to the Hirshfeld surface area of the title compound.

Hirshfeld surface analysis was undertaken in order to better understand the inter­molecular inter­actions within the crystal structure using graphical tools (Spackman & Jayatilaka, 2009 ▸; Spackman *et al.*, 2021 ▸). Hirshfeld surface analysis provides a three-dimensional picture of the inter­molecular inter­actions. These inter­actions can be summarized by using fingerprint plots. The Hirshfeld surface of the title compound mapped over *d*_norm_ is shown in Fig. 3 ▸. The red spots on the surface indicate the presence of atoms in very close proximity to the outside of the surface, the white means that the atoms are in medium proximity while the blue areas are completely devoid of close contacts. The combination of the 3D Hirshfeld surface and the 2D fingerprint plots (Fig. 4 ▸), shows that inter­molecular H⋯H contacts make the main contribution, corresponding to 47.5% of the total Hirshfeld surface (McKinnon *et al.*, 2007 ▸) and that there are short inter­molecular H⋯H contacts where *d*_e_ = *d*_i_ = 1 Å. In the fingerprint plot delineated into C⋯H/H⋯C contacts (27.6% of the total Hirshfeld surface) there are two short spikes. The red spots on the *d*_norm_ surface in Fig. 3 ▸ are due to the H_O_⋯O contacts corresponding to O—H⋯O and O—H⋯N hydrogen bonds. The O⋯H and N⋯H contacts represent 12.4% and 6.1% of the total Hirshfeld surface, respectively, Fig. 5 ▸. These contacts are manifested as sharp spikes at *d*_e_ + *d*_i_ = 1.8 Å for N⋯H and 1.9 Å for O⋯H. Finally, packing cohesion in this structure is also provided by C⋯N and C⋯C inter­actions, which correspond to π–π stacking inter­actions.

## 5. Synthesis and crystallization

All chemicals were commercially available, purchased from Sigma-Aldrich, and used as received without purification. 3-Hy­droxy­benzaldehyde (0.244 g, 2 mmol) and salicyl­aldehyde (0.244 g, 2 mmol) were added to an ethano­lic solution of *o*-phenyl­enedi­amine (0.216 g, 2 mmol). The reaction mixture was stirred for 4 h under reflux at 348 K. The resulting brown solution was cooled in an ice bath. The obtained filtrate was left to evaporate slowly at room temperature, giving after two weeks colorless crystals suitable for single-crystal x-ray diffraction analysis.

## 6. Refinement

Crystal data, data collection and structure refinement details are summarized in Table 2 ▸. All H atoms were located in difference electron-density maps and were treated as riding on their parent atoms with C—H = 0.93 Å, O—H = 0.84 Å and *U*_iso_(H) = 1.2*U*_eq_(C) or 1.5*U*_eq_(O).

## Supplementary Material

Crystal structure: contains datablock(s) global, I. DOI: 10.1107/S2056989023010368/zn2033sup1.cif

Supporting information file. DOI: 10.1107/S2056989023010368/zn2033Isup2.cml

CCDC reference: 2311475

Additional supporting information:  crystallographic information; 3D view; checkCIF report

## Figures and Tables

**Table 1:** Hydrogen-bond geometry (Å, °)

*D*—H⋯*A*	*D*—H	H⋯*A*	*D*⋯*A*	*D*—H⋯*A*
O1—H1⋯O2^i^	0.82	2.04	2.861 (2)	173
O2—H2⋯N1^ii^	0.82	1.89	2.7124 (19)	178
C16—H16⋯N2	0.93	2.55	2.878 (2)	101

Symmetry codes: (i) 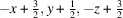 ; (ii) 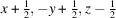 .

**Table 2:** Experimental details

Crystal data	
Chemical formula	C_20_H_16_N_2_O_2_
*M*_r_	316.35
Crystal system, space group	Monoclinic, *P*2_1_/*n*
Temperature (K)	100
*a*, *b*, *c* (Å)	10.6474 (3), 13.2429 (3), 11.4176 (3)
β (°)	99.067 (1)
*V* (Å^3^)	1589.79 (7)
*Z*	4
Radiation type	Mo *K*α
μ (mm^−1^)	0.09
Crystal size (mm)	0.1 × 0.1 × 0.08

Data collection	
Diffractometer	Nonius KappaCCD
Absorption correction	–
No. of measured, independent and observed [*I* > 2σ(*I*)] reflections	33506, 4650, 2866
*R*_int_	0.056

Refinement	
*R*[*F* ^2^ > 2σ(*F* ^2^)], *wR*(*F* ^2^), *S*	0.059, 0.190, 1.06
No. of reflections	4643
No. of parameters	217
H-atom treatment	H-atom parameters constrained
Δρ_max_, Δρ_min_ (e Å^−3^)	0.50, −0.30

Computer programs: *COLLECT* (Nonius, 1999 ▸), *SHELXT* (Sheldrick, 2015*a* ▸), *SHELXL* (Sheldrick, 2015*b* ▸) and *OLEX2* (Dolomanov *et al.*, 2009 ▸).

## supplementary crystallographic information

### Crystal data

**Table:**

C_20_H_16_N_2_O_2_	*F*(000) = 664
*M**_r_* = 316.35	Least Squares Treatment of 25 SET4 setting angles.
Monoclinic, *P*2_1_/*n*	*D*_x_ = 1.322 Mg m^−^^3^
Hall symbol: -P 2yn	Mo *K*α radiation, λ = 0.71073 Å
*a* = 10.6474 (3) Å	Cell parameters from 33506 reflections
*b* = 13.2429 (3) Å	θ = 2.4–30.1°
*c* = 11.4176 (3) Å	µ = 0.09 mm^−^^1^
β = 99.067 (1)°	*T* = 100 K
*V* = 1589.79 (7) Å^3^	Prism, colourless
*Z* = 4	0.1 × 0.1 × 0.08 mm

### Data collection

**Table:**

Nonius KappaCCD diffractometer	2866 reflections with *I* > 2σ(*I*)
Radiation source: fine-focus sealed tube	*R*_int_ = 0.056
Graphite monochromator	θ_max_ = 30.1°, θ_min_ = 2.4°
ω scans	*h* = −15→14
33506 measured reflections	*k* = −17→18
4650 independent reflections	*l* = −15→16

### Refinement

**Table:**

Refinement on *F*^2^	0 restraints
Least-squares matrix: full	Hydrogen site location: inferred from neighbouring sites
*R*[*F*^2^ > 2σ(*F*^2^)] = 0.059	H-atom parameters constrained
*wR*(*F*^2^) = 0.190	W = 1/[Σ^2^(*F*O^2^) + (0.1027*P*)^2^ + 0.1399*P*] WHERE *P* = (*F*O^2^ + 2*F*C^2^)/3
*S* = 1.06	(Δ/σ)_max_ < 0.001
4643 reflections	Δρ_max_ = 0.50 e Å^−^^3^
217 parameters	Δρ_min_ = −0.30 e Å^−^^3^

### Special details

**Table:**

Geometry. Bond distances, angles etc. have been calculated using the rounded fractional coordinates. All su's are estimated from the variances of the (full) variance-covariance matrix. The cell esds are taken into account in the estimation of distances, angles and torsion angles

### Fractional atomic coordinates and isotropic or equivalent isotropic displacement parameters (Å^2^)

**Table:**

	*x*	*y*	*z*	*U*_iso_*/*U*_eq_	
O1	0.71485 (14)	0.50588 (12)	0.85134 (15)	0.0674 (6)	
O2	0.86823 (12)	0.19463 (10)	0.75574 (10)	0.0484 (4)	
N1	0.50090 (14)	0.18753 (11)	1.12414 (12)	0.0415 (4)	
N2	0.64015 (13)	0.13212 (10)	1.01162 (12)	0.0365 (4)	
C1	0.54208 (16)	0.30396 (13)	0.96699 (14)	0.0393 (5)	
C2	0.64184 (18)	0.36170 (13)	0.93853 (15)	0.0434 (5)	
C3	0.61480 (19)	0.45154 (14)	0.87656 (16)	0.0470 (6)	
C4	0.49058 (19)	0.48277 (14)	0.84368 (16)	0.0496 (6)	
C5	0.3922 (2)	0.42579 (16)	0.87286 (17)	0.0524 (6)	
C6	0.41675 (19)	0.33701 (15)	0.93432 (16)	0.0475 (6)	
C7	0.56219 (15)	0.20898 (13)	1.03474 (14)	0.0376 (5)	
C8	0.62905 (16)	0.05631 (12)	1.09273 (15)	0.0386 (5)	
C9	0.54146 (16)	0.09200 (13)	1.16216 (15)	0.0405 (5)	
C10	0.50993 (19)	0.03349 (15)	1.25517 (17)	0.0504 (6)	
C11	0.5675 (2)	−0.05921 (16)	1.27507 (19)	0.0584 (7)	
C12	0.6536 (2)	−0.09453 (14)	1.20405 (19)	0.0573 (7)	
C13	0.68637 (19)	−0.03791 (14)	1.11183 (17)	0.0485 (6)	
C14	0.71836 (15)	0.12707 (14)	0.91676 (14)	0.0389 (5)	
C15	0.85421 (16)	0.16113 (12)	0.95674 (14)	0.0353 (5)	
C16	0.91028 (19)	0.16091 (16)	1.07445 (16)	0.0500 (6)	
C17	1.0337 (2)	0.19263 (17)	1.10898 (18)	0.0565 (7)	
C18	1.10283 (19)	0.22691 (18)	1.02536 (18)	0.0561 (7)	
C19	1.04917 (17)	0.22814 (16)	0.90709 (17)	0.0505 (6)	
C20	0.92539 (16)	0.19490 (12)	0.87220 (14)	0.0364 (5)	
H1	0.69016	0.56161	0.82622	0.1010*	
H2	0.90990	0.22934	0.71643	0.0730*	
H2A	0.72555	0.34062	0.96058	0.0520*	
H4	0.47340	0.54263	0.80161	0.0590*	
H5	0.30871	0.44742	0.85090	0.0630*	
H6	0.34993	0.29882	0.95425	0.0570*	
H10	0.45194	0.05650	1.30205	0.0600*	
H11	0.54868	−0.09918	1.33701	0.0700*	
H12	0.68991	−0.15799	1.21930	0.0690*	
H13	0.74390	−0.06162	1.06486	0.0580*	
H14A	0.68017	0.16920	0.85120	0.0470*	
H14B	0.71891	0.05813	0.88814	0.0470*	
H16	0.86349	0.13880	1.13176	0.0600*	
H17	1.06999	0.19081	1.18856	0.0680*	
H18	1.18584	0.24933	1.04831	0.0670*	
H19	1.09626	0.25138	0.85055	0.0610*	

### Atomic displacement parameters (Å^2^)

**Table:**

	*U*^11^	*U*^22^	*U*^33^	*U*^12^	*U*^13^	*U*^23^
O1	0.0589 (9)	0.0611 (9)	0.0872 (12)	0.0017 (7)	0.0269 (8)	0.0158 (8)
O2	0.0457 (7)	0.0684 (8)	0.0323 (6)	−0.0122 (6)	0.0096 (5)	0.0031 (5)
N1	0.0386 (8)	0.0533 (8)	0.0348 (7)	0.0000 (6)	0.0125 (6)	−0.0014 (6)
N2	0.0342 (7)	0.0432 (7)	0.0342 (7)	−0.0025 (6)	0.0118 (6)	−0.0014 (5)
C1	0.0426 (9)	0.0459 (9)	0.0300 (8)	0.0020 (7)	0.0080 (7)	−0.0044 (6)
C2	0.0446 (10)	0.0482 (9)	0.0383 (9)	0.0035 (8)	0.0092 (8)	−0.0003 (7)
C3	0.0536 (11)	0.0493 (10)	0.0407 (10)	−0.0016 (8)	0.0157 (8)	−0.0028 (7)
C4	0.0579 (12)	0.0499 (10)	0.0395 (10)	0.0095 (9)	0.0034 (8)	0.0003 (8)
C5	0.0495 (11)	0.0612 (11)	0.0446 (10)	0.0083 (9)	0.0012 (8)	−0.0028 (9)
C6	0.0458 (10)	0.0550 (10)	0.0418 (10)	0.0037 (8)	0.0072 (8)	−0.0037 (8)
C7	0.0346 (8)	0.0475 (9)	0.0312 (8)	−0.0019 (7)	0.0068 (6)	−0.0039 (6)
C8	0.0349 (8)	0.0438 (8)	0.0370 (9)	−0.0085 (7)	0.0058 (7)	−0.0033 (7)
C9	0.0365 (9)	0.0489 (9)	0.0371 (9)	−0.0074 (7)	0.0089 (7)	−0.0024 (7)
C10	0.0487 (11)	0.0623 (11)	0.0426 (10)	−0.0139 (9)	0.0146 (8)	0.0028 (8)
C11	0.0643 (13)	0.0582 (12)	0.0519 (12)	−0.0225 (10)	0.0063 (10)	0.0102 (9)
C12	0.0674 (14)	0.0410 (9)	0.0600 (12)	−0.0101 (9)	−0.0006 (10)	0.0029 (9)
C13	0.0506 (11)	0.0445 (9)	0.0505 (11)	−0.0031 (8)	0.0081 (9)	−0.0044 (8)
C14	0.0360 (9)	0.0501 (9)	0.0325 (8)	−0.0039 (7)	0.0117 (7)	−0.0054 (7)
C15	0.0343 (8)	0.0411 (8)	0.0317 (8)	0.0004 (6)	0.0085 (6)	−0.0022 (6)
C16	0.0484 (11)	0.0690 (12)	0.0331 (9)	−0.0117 (9)	0.0076 (8)	0.0055 (8)
C17	0.0517 (12)	0.0776 (14)	0.0362 (10)	−0.0147 (10)	−0.0051 (8)	0.0049 (9)
C18	0.0373 (10)	0.0784 (13)	0.0500 (11)	−0.0128 (9)	−0.0007 (8)	0.0048 (10)
C19	0.0382 (10)	0.0718 (12)	0.0432 (10)	−0.0084 (9)	0.0120 (8)	0.0076 (9)
C20	0.0370 (9)	0.0427 (8)	0.0304 (8)	0.0018 (7)	0.0077 (7)	−0.0008 (6)

### Geometric parameters (Å, º)

**Table:**

O1—C3	1.354 (3)	C14—C15	1.515 (2)
O2—C20	1.372 (2)	C15—C20	1.392 (2)
N1—C7	1.326 (2)	C15—C16	1.382 (2)
N1—C9	1.384 (2)	C16—C17	1.377 (3)
O1—H1	0.8200	C17—C18	1.372 (3)
N2—C7	1.365 (2)	C18—C19	1.381 (3)
N2—C14	1.468 (2)	C19—C20	1.387 (3)
O2—H2	0.8200	C2—H2A	0.9300
N2—C8	1.384 (2)	C4—H4	0.9300
C1—C6	1.398 (3)	C5—H5	0.9300
C1—C2	1.388 (3)	C6—H6	0.9300
C1—C7	1.475 (2)	C10—H10	0.9300
C2—C3	1.391 (3)	C11—H11	0.9300
C3—C4	1.380 (3)	C12—H12	0.9300
C4—C5	1.374 (3)	C13—H13	0.9300
C5—C6	1.373 (3)	C14—H14A	0.9700
C8—C13	1.391 (2)	C14—H14B	0.9700
C8—C9	1.398 (2)	C16—H16	0.9300
C9—C10	1.398 (3)	C17—H17	0.9300
C10—C11	1.375 (3)	C18—H18	0.9300
C11—C12	1.397 (3)	C19—H19	0.9300
C12—C13	1.382 (3)		
			
C7—N1—C9	105.67 (14)	C17—C18—C19	119.99 (19)
C3—O1—H1	109.00	C18—C19—C20	120.34 (18)
C7—N2—C14	127.55 (14)	C15—C20—C19	119.96 (15)
C8—N2—C14	125.38 (14)	O2—C20—C15	117.67 (15)
C7—N2—C8	107.03 (13)	O2—C20—C19	122.37 (15)
C20—O2—H2	109.00	C1—C2—H2A	120.00
C6—C1—C7	117.43 (16)	C3—C2—H2A	120.00
C2—C1—C6	119.90 (16)	C3—C4—H4	120.00
C2—C1—C7	122.65 (15)	C5—C4—H4	120.00
C1—C2—C3	119.05 (17)	C4—C5—H5	120.00
C2—C3—C4	120.49 (18)	C6—C5—H5	120.00
O1—C3—C2	117.14 (18)	C1—C6—H6	120.00
O1—C3—C4	122.37 (17)	C5—C6—H6	120.00
C3—C4—C5	120.27 (18)	C9—C10—H10	121.00
C4—C5—C6	120.21 (19)	C11—C10—H10	121.00
C1—C6—C5	120.08 (18)	C10—C11—H11	119.00
N1—C7—C1	122.56 (15)	C12—C11—H11	119.00
N1—C7—N2	112.15 (15)	C11—C12—H12	119.00
N2—C7—C1	125.24 (14)	C13—C12—H12	119.00
N2—C8—C13	132.39 (16)	C8—C13—H13	122.00
C9—C8—C13	121.99 (16)	C12—C13—H13	122.00
N2—C8—C9	105.62 (14)	N2—C14—H14A	109.00
C8—C9—C10	120.33 (16)	N2—C14—H14B	109.00
N1—C9—C8	109.52 (15)	C15—C14—H14A	109.00
N1—C9—C10	130.13 (16)	C15—C14—H14B	109.00
C9—C10—C11	117.80 (18)	H14A—C14—H14B	108.00
C10—C11—C12	121.29 (19)	C15—C16—H16	119.00
C11—C12—C13	121.90 (18)	C17—C16—H16	119.00
C8—C13—C12	116.69 (18)	C16—C17—H17	120.00
N2—C14—C15	112.94 (13)	C18—C17—H17	120.00
C16—C15—C20	118.44 (16)	C17—C18—H18	120.00
C14—C15—C16	122.54 (15)	C19—C18—H18	120.00
C14—C15—C20	119.02 (14)	C18—C19—H19	120.00
C15—C16—C17	121.63 (18)	C20—C19—H19	120.00
C16—C17—C18	119.63 (19)		
			
C9—N1—C7—N2	0.45 (19)	C3—C4—C5—C6	−0.4 (3)
C9—N1—C7—C1	178.12 (15)	C4—C5—C6—C1	−0.3 (3)
C7—N1—C9—C8	−0.12 (19)	N2—C8—C9—N1	−0.25 (19)
C7—N1—C9—C10	178.39 (19)	N2—C8—C9—C10	−178.92 (16)
C8—N2—C7—N1	−0.62 (19)	C13—C8—C9—N1	179.53 (16)
C8—N2—C7—C1	−178.22 (15)	C13—C8—C9—C10	0.9 (3)
C14—N2—C7—N1	177.32 (15)	N2—C8—C13—C12	179.02 (18)
C14—N2—C7—C1	−0.3 (3)	C9—C8—C13—C12	−0.7 (3)
C7—N2—C8—C9	0.51 (18)	N1—C9—C10—C11	−178.49 (18)
C7—N2—C8—C13	−179.25 (19)	C8—C9—C10—C11	−0.1 (3)
C14—N2—C8—C9	−177.49 (15)	C9—C10—C11—C12	−0.7 (3)
C14—N2—C8—C13	2.8 (3)	C10—C11—C12—C13	0.9 (3)
C7—N2—C14—C15	94.94 (19)	C11—C12—C13—C8	−0.2 (3)
C8—N2—C14—C15	−87.5 (2)	N2—C14—C15—C16	22.2 (2)
C6—C1—C2—C3	−0.6 (3)	N2—C14—C15—C20	−157.17 (15)
C7—C1—C2—C3	−178.60 (16)	C14—C15—C16—C17	−179.68 (19)
C2—C1—C6—C5	0.8 (3)	C20—C15—C16—C17	−0.3 (3)
C7—C1—C6—C5	178.91 (17)	C14—C15—C20—O2	−0.6 (2)
C2—C1—C7—N1	134.27 (18)	C14—C15—C20—C19	178.84 (16)
C2—C1—C7—N2	−48.4 (2)	C16—C15—C20—O2	−179.98 (16)
C6—C1—C7—N1	−43.8 (2)	C16—C15—C20—C19	−0.6 (3)
C6—C1—C7—N2	133.60 (18)	C15—C16—C17—C18	1.0 (3)
C1—C2—C3—O1	179.55 (16)	C16—C17—C18—C19	−0.9 (3)
C1—C2—C3—C4	−0.1 (3)	C17—C18—C19—C20	0.0 (3)
O1—C3—C4—C5	−179.00 (18)	C18—C19—C20—O2	−179.92 (19)
C2—C3—C4—C5	0.6 (3)	C18—C19—C20—C15	0.7 (3)

### Hydrogen-bond geometry (Å, º)

**Table:**

*D*—H···*A*	*D*—H	H···*A*	*D*···*A*	*D*—H···*A*
O1—H1···O2^i^	0.82	2.04	2.861 (2)	173
O2—H2···N1^ii^	0.82	1.89	2.7124 (19)	178
C16—H16···N2	0.93	2.55	2.878 (2)	101

Symmetry codes: (i) −*x*+3/2, *y*+1/2, −*z*+3/2; (ii) *x*+1/2, −*y*+1/2, *z*−1/2.
